# Eating behaviour, quality of life and cardiovascular risk in obese and overweight children and adolescents: a cross-sectional study

**DOI:** 10.1186/s12887-023-04107-w

**Published:** 2023-06-17

**Authors:** Fernanda Gabriela Colombo Drumond Santos, Mariana Godoy-Leite, Eduardo Augusto Resende Penido, Kennad Alves Ribeiro, Maria da Gloria Rodrigues-Machado, Bruno Almeida Rezende

**Affiliations:** Programa de Pós-Graduação em Ciências da Saúde - Faculdade Ciências Médicas- MG, Belo Horizonte, MG Brasil

**Keywords:** Arterial stiffness, Pulse wave velocity, Quality of life, Childhood obesity, Eating behaviour

## Abstract

**Background:**

Childhood obesity is a major cardiovascular risk factor because it predisposes individuals to comorbidities that are implicated in an increased risk of cardiovascular events. Its origin may be related to poor eating habits, such as the intake of foods of low nutritional value or inadequate eating behaviours related to emotional factors. This work aims to evaluate the relationship between the total body mass of children and adolescents and its association with eating habits, quality of life (QoL), and possible changes in early markers of cardiovascular risk.

**Methods:**

This was a cross-sectional observational study that evaluated anthropometric and cardiovascular parameters, QoL, and eating behaviour in 181 children and adolescents aged between 5 and 13 years. Participants were stratified according to BMI/age into three groups (Adequate Weight, Overweight, and Obesity). Anthropometry included weight, height, waist and hip circumferences, waist-hip ratio, and waist-height ratio. QoL was assessed using the Peds-QL 4.0 questionnaire, and eating behaviour was assessed using the Children’s Eating Behaviour Questionnaire (CEBQ). Cardiovascular parameters were assessed using the Mobil-O-Graph® device, which measures the pulse wave velocity (PWV) and augmentation index (AIx@75) to estimate arterial stiffness (AS), which is considered an early marker of cardiovascular disease.

**Results:**

In addition to the increase in anthropometric measurements (p < 0.001), the Obesity group exhibited behaviour related to food intake (p < 0.05). When analysing QoL, a worsening in the social domain was also observed in the Obesity group (p < 0.05). However, PWV and AIx@75 did not differ between groups.

**Conclusions:**

Eating behaviour is related to the development of childhood obesity. However, early markers of cardiovascular risk related to AS did not change as a function of total body mass in the children evaluated.

## Background

In the first years of life, children begin to develop the characteristics of their eating behaviour. These characteristics tend to be reflected in the eating habits of adulthood and are strongly related to the development of chronic diseases throughout life, including diabetes and obesity [1, 2]. Eating behaviour is evaluated by observing individuals’ characteristics, such as their relationship of pleasure with food, food responsiveness, the habit of eating in the absence of hunger, and willingness to seek access to food [3, 4].

Obesity in childhood and adolescence is a cardiovascular risk factor for great impact, as it predisposes individuals to associated comorbidities, such as arterial hypertension, dyslipidaemia, and diabetes, all of which are implicated in an increased risk of cardiovascular events [5]. Identification of obesity should be associated with the investigation of its origin, which may be related to poor eating habits, ingestion of foods of low nutritional value, or inadequate eating behaviour related to emotional factors [3, 4]. Obesity is a condition that can be altered in family nuclei because it is a modifiable factor for cardiovascular risk. Parents’ perception of their children’s dietary education is important in the analysis of repercussions, such as food selectivity, increased intake of sugars, and weight gain [6].

Epidemiological studies have reported an increased incidence of arterial hypertension during childhood and adolescence in recent years, probably due to the association of overweight, obesity, and physical inactivity, which were even more frequent and evident in the COVID-19 period [7]. The current scientific literature proposes an early investigation, still during childhood, of risk factors associated with cardiovascular diseases, such as obesity, and its association with other predictors of cardiovascular risk so that interventions to minimize the chances of cardiovascular disease and the occurrence of cardiovascular events in adulthood can be planned [8–10].

Stiffness of the great arterial vessels has been considered an independent marker of cardiovascular risk, and the findings of atherosclerotic lesions in adolescent patients demonstrate that the process of coronary artery disease begins very early in life [11]. Thus, this study evaluates the relationship between eating habits and their impact on the total body mass of children and adolescents to establish possible associations of these parameters with quality of life (QoL) and early markers of cardiovascular risk. These findings may contribute to a multiple approach aimed at minimizing the development of overweight and obesity and the onset of early morphofunctional changes related to the high incidence of cardiovascular events in the adult population.

## Methods

### Study design

This was a cross-sectional, observational study that evaluated the anthropometric and cardiovascular parameters, QoL, and eating behaviours of children and adolescents.

### Participants

The participants in this study were 181 healthy children and adolescents of both sexes aged between 5 and 13 years old from public schools in the city of Belo Horizonte, Minas Gerais, Brazil, categorized into three groups according to BMI: Adequate Weight, Overweight and Obesity. The body mass index for age (BMI/Age) z score proposed by the World Health Organization (WHO) was used for this categorization [12]. The WHO classifies children into six categories of total body mass according to the z score: marked thinness, thinness, adequate weight, overweight, obesity, and severe obesity. The sample of this study was classified into adequate weight, overweight, and obesity, as shown in Table 1.

**Table 1:** Classification of the study subjects

WHO classification	Number of individuals per subgroup	Number of individuals after grouping	Study group stratification
Marked thinness	0	127	Adequate weight
Thinness	4		
Normal weight	123		
Overweight	38	38	Overweight
Obesity	14	16	Obesity
Severe Obesity	2		

The children were evaluated from March to November 2021. Volunteers who reported acute or chronic cardiovascular and/or renal diseases, respiratory diseases, and diabetes were excluded. The *International Study of Asthma and Allergies in Childhood* (ISAAC) questionnaire was used to investigate possible respiratory diseases; volunteers with a score greater than or equal to five points were excluded [13]. These conditions require exclusion from the study because they may directly affect the results of the cardiovascular evaluation [14–17].

### Anthropometric evaluation

Weight, height, waist and hip circumferences (WC and HC), and waist-hip and waist-height ratios (WHtR) were evaluated. The WHO considers the waist-hip ratio as a criterion to characterize metabolic syndrome and cardiovascular risk, with cutoff values of 0.90 for men and 0.85 for women [18]. For children, however, there is no defined range of values for a favourable waist-hip ratio. In the paediatric population, the waist-to-height ratio seems more reasonable, and studies have demonstrated that a ratio > 0.50 is related to cardiovascular changes and in children with normal weight, indicating a less favourable metabolic profile [19].

### Assessment of quality of life (QoL)

The QoL of the participants was assessed using the Pediatric Quality of Life Inventory version 4.0 (Peds-QL 4.0). This questionnaire comprises 23 questions that assess the perception of children and adolescents in the following dimensions in the past month: physical (eight items); emotional - cognitive and intellectual (five items); social (five items); and school (five items) [20]. The answers to these questionnaires were provided by the parents or guardians together with the participants involved, who were assisted by the study researchers when necessary.

### Assessment of eating behaviour

Eating behaviour was assessed using the Children’s Eating Behaviour Questionnaire (CEBQ) questionnaire, which considers the subjective perception of parents or guardians of the participating children’s eating and behavioural characteristics [3]. This inventory has already been validated for a sample of Portuguese children aged between 3 and 13 years in Portugal [21] and has already been applied to a population of Brazilian children aged between 6 and 10 years [22]. The CEBQ contains 35 questions divided into eight subscales, four of which investigate behaviours that reflect “interest in food” - pleasure in eating (EF), response to food (RF), desire to drink (DD), and emotional intake (EOE) - and four other subscales that reflect behaviours of “disinterest in food” - satiety response (SR), emotional undereating (EUE), selectivity (FF), and slow eating (SE).

The score is assessed using a Likert scale ranging from 1 to 5, as follows: [1] never, [2] rarely, [3] sometimes, [4] often, and [5] always. The subscales have a mean value and standard deviation based on the sum of the scores of the questions belonging to the same subscale. Each subscale is individually smoothed, and there is no total test score. The higher the score for each subscale is, the greater the intensity of the analysed behaviour. The final analysis of the scores was performed according to Viana and Sinde (2008) [21].

#### Evaluation of cardiovascular parameters

Cardiovascular parameters were evaluated according to previous studies by our group [15, 23]. Arterial stiffness (AS) was noninvasively estimated by measuring pulse wave velocity (PWV) and frequency-corrected augmentation index of 75 bpm (AIx@75) using the Mobil-O-Graph® device (IEM Germany - The Pulse Wave Analysis Monitor, version 4.8, Vienna, Austria). This device uses an oscillometric method of assessing brachial arterial pressure to estimate central arterial pressure. The aortic pressure wave was obtained as the sum of the incident pressure waves generated by ventricular contraction and the reflected pressure wave from the periphery. The augmentation pressure (AP) corresponds to the increase in central systolic blood pressure (SBPc) due to the reflection wave, which, when expressed as a percentage of the central pulse pressure (PPc), corresponds to AIx@75 = AP/PPc × 100. The ARSSolver algorithm allows the PWV to be calculated by means of a mathematical model, considering several parameters in the pulse wave and the wave separation analysis [24].

The device also allows the evaluation of several other parameters, including peripheral systolic (SBPp), diastolic (DBPp), mean (MAPp), and pulse (PPp) blood pressures in addition to SBPc, central diastolic blood pressure (DBPc), and PPc. In addition, stroke volume (SV), cardiac output (CO), cardiac index (CI), total vascular resistance (TVR), and heart rate (HR) were also measured.

Similar to the CI, the relationship between SV and body surface area (SB) expressed in square metres was used to allow comparison between different individuals. The exams were performed in the sitting position after at least 10 min of rest. The average of three consecutive records obtained automatically was considered for the final analysis.

#### Sample size

The sample size was calculated to test the difference in the means of AIx@75 of children and adolescents [25], with respect to the classification into adequate weight, overweight, and obesity. At a significance level of 5% and minimum power of 80%, to test a minimum difference of 4.1 in AIx@75 in relation to the mean AIx@75 obtained in a similar study [26], at least 180 individuals were needed.

### Ethical aspects of the research

This study was approved by the Research Ethics Committee of the Faculty of Medical Sciences of Minas Gerais (protocol n. 48326715.5.0000.5134, approval report n. 4,551,378). All parents or guardians provided written informed consent, and the participants agreed to participate in the study by completing an assent form.

### Statistical analysis

The data are presented in tables with absolute frequencies and their respective percentages and with descriptive measures (mean, standard deviation and median) for quantitative data. The quantitative variables were tested for normality using the Kolmogorov‒Smirnov test. As the continuous variables related to the assessed questionnaires did not have a normal distribution, nonparametric tests were used (Kruskal‒Wallis and Mann‒Whitney tests). The significance level adopted was p < 0.05. The software used for the analyses was SPSS version 25.0.

## Results

The sample consisted of 181 volunteers, most of whom were female (56.4%). The mean age was 9.2 ± 2 years (5 to 13 years). In terms of total body mass, the mean BMI was 18.8 ± 4 kg/m^2^ (12.9 to 43.6 kg/m^2^) (Table 2).

**Table 2:** The participants’ sociodemographic data

Parameters	
Sex	
Female	102 (56.4%)
Male	79 (43.6%)
Age (years)	9.2 ± 2
Weight (kg)	37.8 ± 12.7 (35)
Height (cm)	140 ± 13 (140)
BMI (kg/m^2^)	18.8 ± 4 (18)
WC (cm)	66.5 ± 11.2 (64)
HC (cm)	78.8 ± 11 (78)
WC/HC WHtR	0.85 ± 0.1 (0.8) 0.47 ± 0.07 (0.5)

The data are expressed as the mean ± standard error of the mean (median), except for sex (expressed as n and percentage). BMI: body mass index, WC: waist circumference, HC: hip circumference, WHtR: waist-to-height ratio

Table 3 shows that the anthropometric variables of the Overweight and Obesity groups did not differ and were significantly higher than those of the Adequate Weight group.

**Table 3:** Anthropometric parameters according to the study groups

Anthropometric variables	Total Body Mass (BMI/Age)			
	Adequate Weight (n = 127)	Overweight (n = 38)	Obesity (n = 16)	p value
Percentile BMI/age	52.6 ± 26.6 (59)	90.7 ± 13.8 (93)*	98.5 ± 0.9 (99)*	< 0.001^k^
WC (cm)	61.7 ± 6.4 (62)	74 ± 9.5 (86)*	85.7 ± 14.5 (85)*	< 0.001^k^
HC (cm)	75.1 ± 7.7 (75)	85 ± 9.6 (84)*	92.8 ± 17.5 (93)*	< 0.001^k^
WC/HC	0.8 ± 0.1 (0.8)	0.9 ± 0.1 (0.9)*	0.9 ± 0.3 (0.3)*	< 0.001^k^
WHtR	0.4 ± 0 (0.4)	0.5 ± 0.1 (0.5)*	0.6 ± 0.1 (0.1)*	< 0.001^k^

The data are expressed as the mean ± standard error of the mean (median), except for sex (expressed as n and percentage). BMI: body mass index, WC: waist circumference, HC: hip circumference, WHtR: waist-to-height ratio, * p < 0.05 compared to the Adequate Weight group. ^k^ Kruskal‒Wallis test

Evaluation of QoL by the PEDS revealed that the social domain was significantly lower in the Obesity group than in the Adequate Weight group (p < 0.05) (Table 4).

In terms of eating behaviour, evaluated by the CEBQ, the Satiety Response was significantly lower (p < 0.001) and the Response to Food (p < 0.001), Pleasure in Eating (p < 0.05), Emotional Overeating (p < 0.001), and Desire to Drink (p < 0.05) domains were significantly higher in the Obesity group than in the Adequate Weight group. The Emotional Overeating domain was also higher in the Obesity group than in the Overweight group (p < 0.05) (Table 4).

**Table 4:** Life quality and eating behaviour according to the study groups

				Total Body Mass (BMI/Age)					
Questionnaire	Domain		Adequate Weight (n = 127)		Overweight (n = 38)	Obesity (n = 16)	p value*		
PEDS	Physical		81.99 ± 16.18 (84)		79.62 ± 16.72 (87)	75.52 ± 14.72 (75)	0.155		
	Emotional		72.28 ± 19.53 (70)		70.26 ± 22.56 (72)	69.25 ± 18.38 (72)	0.795		
	Social		85.20 ± 16.04 (90)		83.03 ± 16.96 (85)	75.31 ± 14.41 (75)*	**< 0.05**		
	School		74.49 ± 18.12 (75)		69.61 ± 18.11 (70)	68.44 ± 15.38 (70)	0.121		
CEBQ	Satiety Response		2.44 ± 0.75 (2.4)		2.08 ± 0.65 (2.0)	1.98 ± 0.51 (2.1)	**< 0.001**		
	Slowness in Eating		2.56 ± 0.84 (2.5)		2.38 ± 0.91 (2.2)	2.19 ± 0.89 (2.0)	0.113		
	Food Fussiness		2.74 ± 0.81 (2.8)		2.78 ± 0.77 (2.7)	2.82 ± 0.91 (2.7)	0.995		
	Emotional Undereating		2.10 ± 0.82 (2.0)		2.04 ± 0.77 (2.0)	1.98 ± 0.57 (2.0)	0.919		
	Food Response		2.41 ± 1.07 (2.2)		2.86 ± 1.05 (2.9)	3.66 ± 1.27 (4.0)*	**< 0.001**		
	Enjoyment of Food		3.55 ± 0.95 (3.5)		3.99 ± 0.68 (4.0)	4.09 ± 0.9 (4.5)*	**< 0.05**		
	Emotional Overeating		2.06 ± 0.94 (2.0)		2.38 ± 1.19 (2.1)	3.45 ± 1.12 (3.5)*^#^	**< 0.001**		
	Desire to Drink		2.86 ± 1.25 (2.7)		3.16 ± 1.12 (2.8)	3.44 ± 1.34 (3.5)*	**< 0.05**		

The data are expressed as the mean ± standard error of the mean (median). *p values in relation to the Adequate Weight group (Kruskal‒Wallis test). ^#^ p value in relation to the Overweight group (Mann‒Whitney test). Children’s Eating Behaviour Questionnaire (CEBQ), Pediatric Quality of Life Inventory (PEDS)

**Fig. 1:**
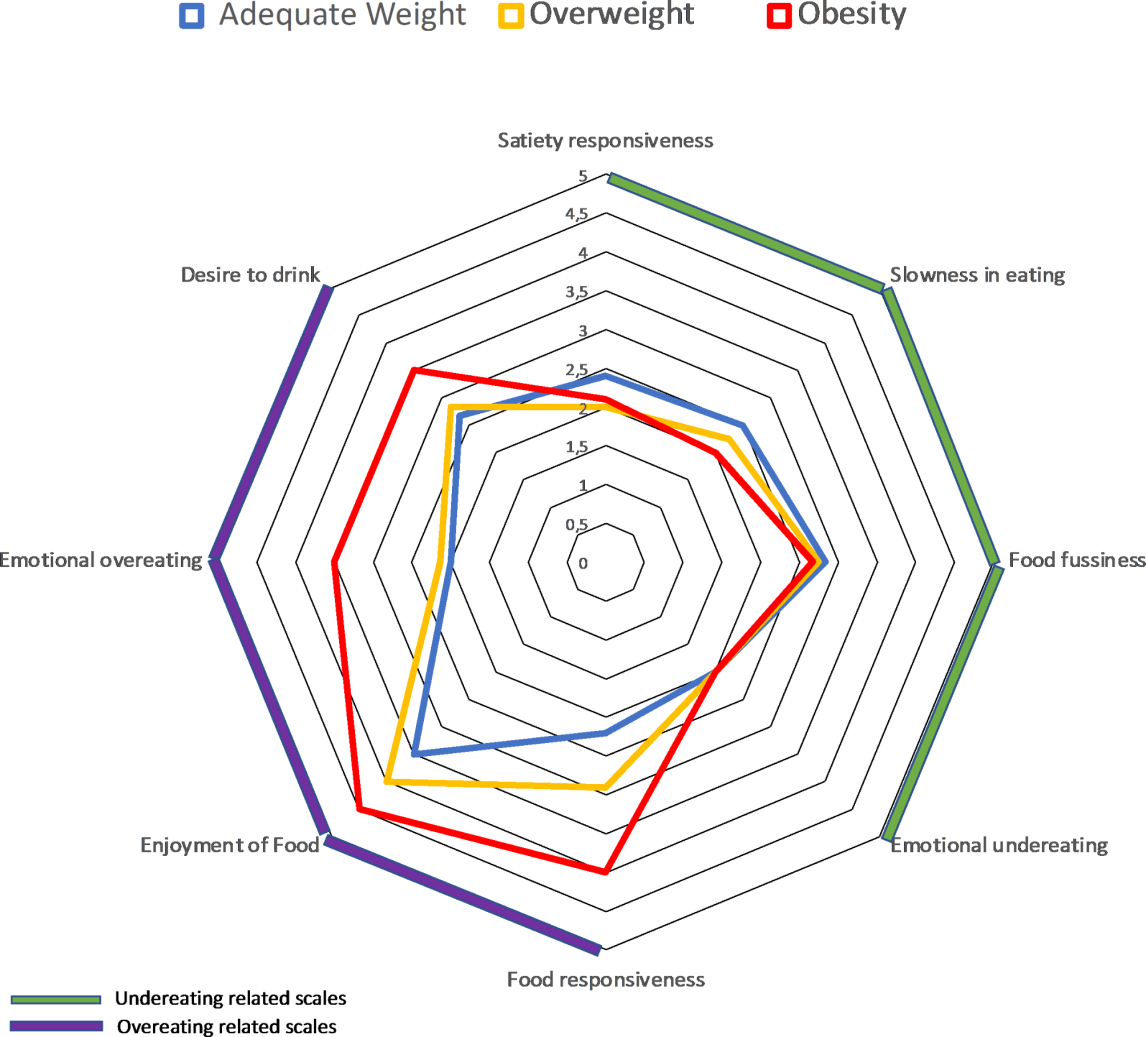
Scores for the subscales of the Children’s Eating Behaviour Questionnaire (CEBQ) according to categories of body mass index/age of the children

Figure 1 is a polar graph that shows the association between the study groups and the eating behaviour questionnaire (CEBQ) scales. The Obesity group had higher scores for the domains related to Overeating (interest in food), i.e., the Response to Food, Pleasure in Eating, Emotional Overeating, and Desire to Drink domains.

The variables of peripheral and central arterial pressure and indices of AS did not differ between the groups. In contrast, the CI was significantly lower in the Obesity group than in the Adequate Weight group (p < 0.01) (Table 5).

**Table 5:** Measurements of peripheral and central blood pressure, haemodynamic parameters, and arterial stiffness of the individuals in the sample, according to the study groups

Variables	Total Body Mass (BMI/Age)			
	Adequate Weight (n = 127)	Overweight (n = 38)	Obesity (n = 16)	p value
	Mean	Mean	Mean	
Peripheral blood pressure				
SBP (mmHg)	109.35 ± 15 (109)	110.78 ± 13 (110)	107.93 ± 10 (108)	0.687^k^
DBP (mmHg)	64.33 ± 13 (64)	66.78 ± 9 (66)	63.12 ± 9.0 (66)	0.421^a^
MAP (mmHg)	84.97 ± 13 (85)	87.00 ± 9 (87)	83.68 ± 8.0 (65)	0.410^a^
PP (mmHg)	45.01 ± 12 (44)	44.00 ± 13 (42)	44.93 ± 11 (45)	0.851^k^
Central blood pressure				
SBP (mmHg)	96.40 ± 14 (96)	98.28 ± 11 (97)	95.12 ± 8 (96)	0.57^k^
DBP (mmHg)	66.36 ± 13 (66)	68.73 ± 9 (68)	65.12 ± 9 (68)	0.409^k^
PP (mmHg)	30.03 ± 8 (29)	29.55 ± 9 (29)	30.00 ± 7 (30)	0.973^k^
Haemodynamics				
VS (ml)	47.43 ± 9.8 (45)	48.33 ± 7.7 (48)	49.10 ± 10 (46)	0.675^k^
CO (l/min)	4.31 ± 0.6 (4.3)	4.49 ± 0.5 (4.4)	4.59 ± 0.8 (4.5)	0.142^a^
TVR (s*mmHg/ml)	1.19 ± 0.15 (1.2)	1.17 ± 0.1 (1.2)	111 ± 0.16 (1.1)	0.160^k^
CI (l/min.l/m^2^)	3.87 ± 0.8 (3.9)	3.53 ± 0.7 (3.4)	3.25 ± 0.6 (3.4)*	**0.01** ^**k**^
HR (bpm)	90.42 ± 12 (92)	93.65 ± 13 (94)	92.00 ± 10 (92)	0.311^k^
Arterial Stiffness				
PPA	1.51 ± 0.2 (1.48)	1.51 ± 0.2 (1.48)	1.50 ± 0.17 (1.5)	0.94^k^
AIx@75 (%)	30.98 ± 11 (31)	30.00 ± 9.0 (31)	28.00 ± 8 (29)	0.419^k^
PWV (m/s)	4.45 ± 0.5 (4.4)	4.51 ± 0.4 (4.5)	4.39 ± 0.3 (4.4)	0.632^k^

a: ANOVA test; k: Kruskal‒Wallis test. The data are expressed as the mean ± standard error of the mean (median). Systolic blood pressure (SBP), diastolic blood pressure (DBP), mean arterial pressure (MAP), pulse pressure (PP), pulse pressure amplification (PPA), augmentation pressure (AP), stroke volume (SV), cardiac output (CO), cardiac index (CI), total vascular resistance (TVR), heart rate (HR), pulse wave velocity (PWV), and augmentation index normalized to 75 bpm (AIx@75). * p value in relation to the Adequate Weight group

## Discussion

Our data demonstrate that obese children manifest behaviour related to overintake. When analysing QoL, a worsening in the social domain was also observed in obese children. However, the haemodynamic parameters and variables related to the development of AS did not differ between the groups.

All domains related to overeating were significantly increased in obese children. The domains related to underingestion, in turn, tended to be lower in this same group than in children in the Adequate Weight or Overweight groups, but with a significant difference only for the domain Response to Satiety. Very similar results were reported in two studies that also evaluated Brazilian children using the same instrument and stratified by BMI [22, 27]. Emotional Overeating, which is characterized by increased consumption of foods related to psychic states with emotional characteristics such as anxiety, anger, and fear, was higher in the Overweight and Obesity groups than in children with adequate weight. Emotional Undereating, in turn, was not altered. This result was also observed in the studies by Passos et al. (2015) and Webber et al. (2009) [22, 28]. In addition, the ingestion of food in states of an emotional lack of control tends to favour the predilection for sweet or fatty foods, thus markedly increasing calorie consumption [29]. Thus, an increased score for the Over Emotional Intake scale should be seen as worrisome due to its close relationship with obesity. In addition, the desire for sugary drinks, which can be assessed in the Desire to Drink domain, may also be related to these emotional factors. The psychosomatic theory of obesity, which identifies the frequent consumption of high-calorie foods as a coping mechanism for emotional problems, could explain these findings [30].

In obese children, we also observed an increase in the Pleasure in Eating scale, which relates to the sensation of well-being during eating, and the Response to Food scale, which refers to external influences of food, such as aroma, texture, and flavour. Both scales assess interest in food [22]. Studies have found that obese children have great interest in food, especially processed foods with striking colours and aromas, in packaging with a strong visual component, strategically developed to arouse interest in such products [31, 32]. For this reason, several entities have tried to create ways to combat this marketing strategy related to these foods as a means of reducing the incidence of childhood obesity.

It was already expected that eating behaviour would be closely related to the development of obesity. An objective evaluation of eating behaviour is necessary mainly because the early detection of these behaviours could lead to measures to prevent the development of obesity and overweight. The coincidence of results from different studies using the CEBQ questionnaire reinforces the efficiency of the instrument in determining eating habits and its close association with the total body mass of children in the studied age group.

All domains of the QoL questionnaire progressively worsened with increasing body mass index and were significantly worse in the Social domain for obese children than those in the Adequate Weight group. The literature has already shown that QoL is strongly affected in both obese and overweight children and adolescents. In a meta-analysis on the subject, Buttitta et al. (2014) showed that in 34 articles surveyed using various QoL questionnaires, including the PedsQL-4, only three did not report worsening of QoL in obese paediatric patients [33]. In the same study, the authors showed that the Social and Physical domains were the most affected domains. These results were confirmed in a recent study that, in addition to indicating worsening mainly in the Social and Physical domains, also showed that these results are independent of whether the questionnaires are answered by the children themselves or their guardians [34]. Additionally, Pakpour et al. (2019) demonstrated a direct relationship of worsening in the Physical and Social domains as BMI increased [35].

Both obesity and QoL are already widely discussed as risk factors for the development of cardiovascular diseases in the adult population and even in the paediatric population [36]. However, unlike in the adult population, cardiovascular risk is not routinely assessed in the paediatric population. Recently, some studies have attempted to find markers of cardiovascular risk in the paediatric population, as vascular disease can begin in early childhood and remain asymptomatic in a subclinical manner until it manifests itself in adulthood [11]. Some studies have evaluated non-invasive arterial stiffness markers in the young population due to the high predictive value of this variable for the development of cardiovascular diseases [17, 37]. The values of central arterial pressure measured with the Mobil-O-Graph® device are accurate in children and may show promise as markers of cardiovascular risk in the paediatric population [37]. Thus, we sought to assess whether the haemodynamic variables and arterial stiffness were worse in children with higher BMI values. The identification of the early stages of cardiovascular damage and the recognition of risk factors at a time when these processes are still reversible, as well as the development of prevention strategies, could be major pillars in the reduction of morbidity and mortality from cardiovascular diseases in the general population.

However, when analysing these parameters, we were unable to identify important differences in the haemodynamic variables and AS when classifying the children according to total body mass. Corroborating the data by Castro et al. (2016) [38], the only variable that was different was the CI, which was lower in obese children than in children with adequate weight. As we did not find changes in CO and its components SV and HR, this finding is possibly related to variations in total body mass [38]. The reduction in CI may be associated with poor tissue perfusion, resulting from critical changes in cardiac function in obese children as a result of variations in total body mass [38].

In children, some studies associate the parameters of arterial stiffness with total body mass. Castro et al. [38] also used the Mobil-O-Graph® device and found changes in obese children both in haemodynamic parameters such as an increase in pulse pressure and central systolic pressure and in PWV, the main parameter associated with arterial stiffness. However, in the same study, they evaluated the PWV measured by tonometry and found no difference between the groups. Another recent study found a positive correlation between PWV and BMI in children [39]. Santos et al. (2021) showed a positive correlation between PWV and BMI but an inverse correlation between AIx@75 and BMI; they concluded that BMI and anthropometric measurements would not be good predictors of arterial stiffness. The limited data in the literature evaluating parameters related to arterial stiffness in children are therefore still conflicting. Initially, we expected to find differences in the main haemodynamic parameters in our study, but the absence of significant changes makes us question whether the evaluation of these parameters could truly be used as a marker of cardiovascular risk in this population.

It is very important to emphasize that our work was focused on the evaluation of eating behavior, which is just one of the factors related to the development of obesity and overweight. Other no less important factors such as sedentary lifestyle, the genetic and endocrinological component should also be investigated, as we know that obesity is multifactorial. Today, in our Western society, obesity appears as one of the most stigmatizing and least socially acceptable conditions among children, which can lead obese children to form a negative self-assessment of themselves [40]. It is important not only to be concerned with the factors related to the development of obesity, but also to prepare ourselves to help obese children to overcome their psychological, emotional and social difficulties.

### Study limitations

We used BMI as a measure to interpret the weight change. Body composition can be highly variable and still produce the same BMI. In addition, BMI does not provide information on the regional distribution of body fat. Another limitation of the study was the fact that the evaluations were performed at a critical time during the COVID-19 pandemic, and we do not know whether the children evaluated were infected with COVID-19 and whether the infection would have any impact on the results obtained.

## Conclusions

Our results suggest that obese children and adolescents have typical characteristics of eating behaviours related to overeating and decreased QoL. However, haemodynamic parameters and the evaluation of arterial stiffness were not different in obese or overweight children and adolescents compared to children with adequate weight, which leads us to question the applicability of these parameters in identifying the initial stages of cardiovascular damage related to obesity.

## Data Availability

The dataset analyzed during the current study is available from the corresponding author upon reasonable request.

## List of abbreviations

AS: Arterial stiffness
AIx@75: Augmentation index corrected by the heart rate of 75 bpm
AP: Augmentation pressure
BMI: Body mass index
CBEQ: Children’s Eating Behaviour Questionnaire
CI: Cardiac Index
CO: Cardiac output
DBP: Diastolic blood pressure
HC: Hip circumference
HR: Heart rate
IPAQ-C: Physical Activity Questionnaire-Child
ISAAC: International Study of Asthma and Allergies in Childhood
OR: Odds ratio
Peds-QL 4.0: Pediatric Quality of Life Inventory Version 4.0
PP: Pulse pressure
PPA: Pulse pressure amplification
PPc: Central pulse pressure
PPp: Peripheral pulse pressure
PWV: Pulse wave velocity
QoL: Quality of life
SBPc: Central systolic blood pressure
SV: Stroke volume
TVR: Total vascular resistance
WC: Waist circumference
WHO: World Health Organization
WHtR: Waist-height ratio
